# Artificial Immunity1Read before the Medical Society of the State of New York, Albany, February 6, 1894.

**Published:** 1894-04

**Authors:** Henry Reed Hopkins

**Affiliations:** 433 Franklin Street; Buffalo, N. Y.


### ARTIFICIAL IMMUNITY.[1]

BY HENRY REED HOPKINS, M. D., Buffalo, N. Y.

The traditions and the history of the art of medicine have ever asserted the fact of the remarkable exemption or immunity of certain races or individuals from attacks of communicable disease to which their less favored neighbors were liable. The findings of the science of medicine in her investigations and experiments upon the reactions of various contagia upon the lower animals reveals the existence of a similar variation in immunity, proclivity, susceptibility, and is already well advanced in the most ambitious and far-reaching of human endeavor, the attempt to discover the secret of the immune, in order to possess the God-like power of extending his inestimable blessings and advantages to his less-favored brother, the susceptible.

With truth and soberness, it may be said that there does not exist, and there never has been, a more attractive, a more potential scientific investigation, than the study of artificial immunity.

To the elucidation of this problem, medicine is at this moment devoting her highest art and her most profound science—her most creative enthusiasm, and her most cautious painstaking and accurate technique.

Let us recall some of the primitive conceptions embraced in this consideration. The well-attested observation that certain races, families or individuals possessed a peculiar exemption from the attacks of a given disease; for instance, the dark-skinned races from ague, led our forefathers to recognize the condition which we know as immunity—that the individual who was immune to a given disease, was to that form of illness disease-proof. Continuous and contemporaneous with these interesting observations ran a similar set, resulting in the conclusion that certain individuals had the misfortune to be the pathological antithesis of the previous class, differing from the average of their kind in the opposite

---

1. Read before the Medical Society of the State of New York, Albany, February 6, 1894.

direction, and their situation or relation to disease the fathers were wont to indicate by the terms—proclivity, susceptibility.

Here it is worth noting, for the observation may be found of some little significance, that as the fathers recognized instances of wide variation from the average reaction of individuals to acute disease, they also noted that individuals frequently reacted singularly to the operations of certain well-known remedies; that in one class the usually efficient dose would be found utterly inefficient, while in the opposite class extreme or poisonous conditions or effects would result from the exhibition of what was usually considered perfectly harmless and suitable doses. These interesting facts are still grouped about that most fascinating and puzzling term, idiosyncrasy.

The prosecution of the theory of the bacterial origin of infectious disease has brought us many important new truths; not the least of these in interest and suggestiveness is the fact of the communicability of many of our infectious diseases to our neighbors, the lower animals. Had the observation not extended beyond this point, it still must have been regarded as one of the utmost practical importance and suggestiveness, but already the party of exploration is sending back news from much higher altitudes, and we hear from many laboratories that the same veins, the rich ore-bearing veins—immunity, proclivity, susceptibility—known for ages to run among the children of men, are also found, and in a more marked degree in the lower animals. Moreover, these veins, as seen in the lower animals, are workable, are being worked, and the precious metals, the silver and gold of scientific therapeutics, laid up in these everlasting hills of the bacteriologist,—the rabbit, the guinea-pig and the mouse,—are now in sight; in fact, the metallic bars are now in the assayer's office, or on the way to the mint, waiting for the final test and stamp as coin of the realm of scientific medicine.

The questions which the bacteriologist would answer in this investigation: (1.) What is the nature of the change wrought in the individual, child or adult, by an attack of disease,—scarlet fever, small-pox, or vaccinia,—whereby the individual ceases to furnish the requisite culture media for the maintenance and growth of that particular bacterium known to cause, or to be most intimately associated with the cause, of the particular disease? (2.) How far does this principle of immunity, natural or acquired, extend among the infectious diseases? (3.) How far may we extend the condition of artificial immunity for the cure or prevention of disease?

A slight knowledge of the rapidly increasing literature of this subject convinces the writer that satisfactory progress is being made along the lines of each of these enquiries. Regarding the character of natural immunity, perhaps the progress has been the most disappointing, but even in this direction valuable observations or discoveries are reported. We may so consider the observations of Nuttall, Buchner and others upon the aseptic or anti-infectious property of the blood serum of the immune animal, this quality having been found in several instances to be in direct proportion to the degree of the individual's immunity.

Most intimately related to this thought is the discovery of Koch, as to the different methods by which bacterial invasion produces disease, methods which he proposes to recognize and distinguish by use of the terms, bacterial infection and bacterial intoxication. In bacterial infection, the microörganism, for instance, the bacillus of anthrax, gains admission to the body of the susceptible individual, and very soon rapidly increases in numbers, and literally swarms throughout the entire body, invading every organ and tissue. Such microörganisms appear to be inimical to their host wholly or in the main in a mechanical or negative manner; mechanically by blocking the capillaries, and thereby arresting the circulation of the blood and consequent functional activity, and negatively by robbing the tissues of nutrition used in their own upbuilding, particularly of oxygen. The method of attack in cases of bacterial intoxication is quite different from the foregoing, and introduces new and most important characters, ptomaines and tox-albumins, which may be indicated by the single word toxines. Tetanus, typhoid fever, diphtheria, cholera, and possibly tuberculosis, are instances of disease produced by bacterial intoxication.

In these diseases the method of invasion may be the same as before, but the behavior of the microörganism of the disease, from the moment of invasion, differs widely in the following respects: in bacterial infection the microörganisms tend to increase and multiply rapidly, to go and to grow anywhere and everywhere; in bacterial intoxication the microörganisms tend from the first to grow only on certain tissues and in certain localities, to produce local disease, but with their growth they elaborate a virulent poison, which gains admission to the blood, circulates with the blood, and by doing the work of an irritant poison upon the sensitive and susceptible tissues of the various organs of the body, or some of them, produces the given disease.

These poisons of bacterial origin are of two kinds,—ptomaines and tox-albumins; the former are crystalline, alkaloidal bodies, the latter are colloidal. The important role these animal poisons are likely to play in our knowledge of and management of the infection, the preventable diseases, we may get a hint of when we hear that although of bacterial origin, they may be produced at will outside of the body, and yet when introduced into the susceptible individual, with or without their particular microörganisms, they invariably produce their specific diseases, each after his kind.

The possession of knowledge of the precise kind, as to the method of the production of bacterial disease, as indicated by the above statement, has resulted in a decided advance of our thought relating to artificial immunity, and we now know that an animal may have immunity of two distinct kinds,—namely, from bacterial infection and from bacterial intoxication; and our experimenters seem to have demonstrated that an individual may have one without the other, and that in the near future infectious diseases are to be treated or prevented by an increase and right use of our knowledge of both these processes.

Results of experiments bearing upon the condition bacterial intoxication seem to be the most important and suggestive, and appear to establish the following facts: that there resides in the blood serum a chemical substance or substances of vital origin, by some called defensive proteids or anti-toxines, which substances possess the power of rendering pathogenically inert and harmless the ptomaines and tox-albumins, the disease-producing agents, in all cases of bacterial intoxication. That the organism has the ability of producing these defensive proteids or anti-toxines, as their peculiar services are required for the protection of the individual, as seen in Pasteur's treatment for hydrophobia, until their presence and protective activity in the blood, in much more than that of normal ratio, is demonstrable. When an individual is by suitable treatment brought to such a state as to receive with impunity that which is ordinarily a fatal dose of a given ptomaine or tox-albumin, the individual is said to be poison-proof. And to render the susceptible poison-proof, whereby he ceases to be susceptible and becomes immune, is today the highest ambition of the art and the science of medicine.

Information regarding this most interesting condition of artificial immunity,—poison-proof,—points to the fact, as we just observed, that it may be acquired, and that when present, either by Nature or by acquirement, the fortunate possessor, if a female, may transmit the same to her offspring, and what seems to be most fortunate, the milk of the poison-proof mother renders the young almost equally immune. Experiments also indicate that the blood of the poison-proof animal has high curative powers in cases of the given disease occurring in his own kind or in man. Observers also seem to be quite in accord in this testimony, as to the complete individuality of the action of the various toxines. An animal, poison-proof against a given disease,—tetanus, diphtheria, erysipelas or pneumonia,—has thereby not the slightest protection against any other disease.

The writer is well aware of the fact that medical enthusiasts usually have early cause to regret their enthusiasm, and that the rôle of prophet is not popular in this, the closing years of the nineteenth century, and yet ventures to proclaim with enthusiasm the dawn of a new era in medicine, when from a truer knowledge of temperament, proclivity, susceptibility, idiosyncrasy, immunity and artificial immunity—the art of the treatment of disease and the prevention of disease will attain such eminence, and accomplish such results, as heretofore have been attained and accomplished only in dreams.

433 Franklin Street.

---

Points for Trained Nurses.—At the training schools for nurses no applicants are accepted who are under twenty-one years of age or over thirty-five; twenty-five is the preferred age. When application is made by letter it must be addressed to the superintendent of the school. In reply she will receive a circular stating that a personal interview is desirable. If that is impossible, the applicant should write again, saying so and asking for an application blank. This blank must be filled out in the applicant's own handwriting and returned to the superintendent, together with a physician's certificate of health, a letter from a clergyman and the addresses of three women, not relatives, who have known the applicant for several years. These applications are filed, and when a vacancy occurs the most desirable applicant is selected by the president, and is taken for a month on trial. During this month of probation, she will, at almost all the training schools, receive her board and lodging. At the end of the month she may be accepted or rejected as a pupil nurse, and the decision is final.—February *Ladies' Home Journal.*

